# Voxel-Based State Space Modeling Recovers Task-Related Cognitive States in Naturalistic fMRI Experiments

**DOI:** 10.3389/fnins.2020.565976

**Published:** 2021-05-06

**Authors:** Tianjiao Zhang, James S. Gao, Tolga Çukur, Jack L. Gallant

**Affiliations:** ^1^Program in Bioengineering, University of California, Berkeley, Berkeley, CA, United States; ^2^Helen Wills Neuroscience Institute, University of California, Berkeley, Berkeley, CA, United States; ^3^Department of Electrical and Electronics Engineering, Bilkent University, Ankara, Turkey; ^4^National Magnetic Resonance Research Center (UMRAM), Bilkent University, Ankara, Turkey; ^5^Neuroscience Program, Sabuncu Brain Research Center, Bilkent University, Ankara, Turkey; ^6^Department of Psychology, University of California, Berkeley, Berkeley, CA, United States

**Keywords:** functional magnetic resonance imaging, state space, dimensionality reduction, naturalistic stimuli, complex task environments

## Abstract

Complex natural tasks likely recruit many different functional brain networks, but it is difficult to predict how such tasks will be represented across cortical areas and networks. Previous electrophysiology studies suggest that task variables are represented in a low-dimensional subspace within the activity space of neural populations. Here we develop a *voxel-based state space modeling* method for recovering task-related state spaces from human fMRI data. We apply this method to data acquired in a controlled visual attention task and a video game task. We find that each task induces distinct brain states that can be embedded in a low-dimensional state space that reflects task parameters, and that attention increases state separation in the task-related subspace. Our results demonstrate that the state space framework offers a powerful approach for modeling human brain activity elicited by complex natural tasks.

## Introduction

To maximize efficiency and statistical power, most neuroimaging experiments use simple parametric designs and highly focused data analysis. However, the results of such experiments often do not generalize to the real world (Wu M. C. et al., 2006; Krakauer et al., 2017; Yarkoni and Westfall, 2017; Matusz et al., 2019). To address this problem, some neuroimaging experiments use more complex naturalistic conditions, such as watching movies (Hasson et al., 2004; Nishimoto and Gallant, 2011; Huth et al., 2012), listening to stories (Huth et al., 2016), or playing video games (Mathiak and Weber, 2006; Spiers and Maguire, 2007; Mathiak et al., 2011). Neuroimaging data collected under naturalistic conditions elicit complex, dynamic patterns of brain activity across multiple functional networks that reflect the explicit and implicit task structure of the experiment (Çukur et al., 2013b). Therefore, such experiments dramatically increase the complexity of data analysis and modeling. For example, while watching movies selective attention to one target or another may change the representation of information in relevant functional networks (Çukur et al., 2013b), and while playing a video game the dynamic evolution of goals and subgoals over the course of the game might evoke activity in distinct functional networks over time (Spiers and Maguire, 2006). Current methods for describing functional networks rely overwhelmingly on functional connectivity (FC) (Friston, 1994). FC analysis has become popular for use in resting state studies, where the factors driving brain activity are unknown latent variables (Shen et al., 2010; Van Dijk et al., 2010; Yeo et al., 2011; Blumensath et al., 2013; Buckner et al., 2013; Smith et al., 2013; Gordon et al., 2014). However, networks recovered by FC have no clear functional assignment. Therefore, there is a need for new methods of analysis that can efficiently describe whole-brain activity under complex naturalistic conditions.

Previous neurophysiology studies suggest that the population activity vector of a neural population represents task-related information, and that this information can be recovered even when the activities of individual neurons cannot be well-explained (Fitzpatrick et al., 1997; Pouget et al., 2000; Reich et al., 2001; Pillow et al., 2008). Indeed, many neurophysiology studies have treated the activity of multiple neurons as a dynamical system whose state represents task variables. For example, in arm reach tasks, motor neurons in the non-human primate brain traverse uniquely identifiable trajectories in their state space during reaches to distinct targets (Srinivasan et al., 2006; Wu M. C. et al., 2006; Wu W. et al., 2006). In decision making tasks, the state of prefrontal neurons in non-human primates represents several task-relevant variables including the attentional target (Mante et al., 2013). In navigation tasks, the state of parietal neurons in rats represent information about upcoming turns and planned routes (Harvey et al., 2012). In a goal-directed navigation task, the state of neurons in the retrosplenial cortex (RSC) in rats represents cues and reward locations (Vedder et al., 2017). In both rats and non-human primates, the state of a population of neurons also encodes information about a choice that the animal made (Harvey et al., 2012; Mante et al., 2013; Vedder et al., 2017).

These studies provide strong evidence, from both non-human primates and rodents, that task variables are systematically embedded in a task-related state space that is distributed across neural populations. The dimensionality of this task-related state space appears to be markedly lower than the dimensionality of the total activity space spanned by the population activity vector (Harvey et al., 2012; Mante et al., 2013; Vedder et al., 2017). For example, in a rat navigation task, a 3-dimensional subspace of the total activity space of 65 neurons is sufficient to distinguish choices made by the animal (Harvey et al., 2012). In non-human primates, task variables and the animals’ decisions are encoded a 4-dimension subspace of hundreds of neurons (Mante et al., 2013). It is thus likely that the human brain also represents task variables in a low-dimensional subspace of its total activity space. Furthermore, state-space approaches may be particularly useful for understanding brain function in complex, naturalistic tasks that activate complex networks of brain areas (Spiers and Maguire, 2007; Huth et al., 2016). Therefore, analyzing data in the context of the population activity vector may offer new insights about task-related representations in the human brain.

Here we develop a *voxel-based state space modeling* method for analyzing fMRI data under naturalistic conditions. Our framework is inspired by methods developed originally to model primate electrophysiology data (Mante et al., 2013). The framework is based on the idea that task variables, such as stimulus information and cognitive states of the subject, are represented implicitly in the population activity vector of the entire cortex. The framework specifies a simple algorithm that finds the subspace of the entire activity space that best represents these task variables. We use this framework to successfully recover and interpret task-related state spaces in two naturalistic fMRI experiments: a controlled visual attention task and an open-ended video game.

## Materials and Methods

### Experimental Paradigm

#### Subjects

Seven healthy volunteers (six males, one female; ages 25–32) with normal or corrected to normal vision participated in the experiments. Six subjects (S1–S6) participated in the visual search experiment that was published as a part of a previous study (Çukur et al., 2013b). Two male volunteers (age 25 and 26) participated in the second video game experiment that was run as a part of a pilot experiment for a separate purpose (subjects S1 and S7). The experimental procedures were approved by the Institutional Review Board at the University of California, Berkeley. Written informed consent was obtained from all subjects.

#### Experiment Procedure

Subjects participated in two experiments, a visual attention task and a first-person shooter video game. We used the visual attention task to investigate whether representations of known task variables in a relatively controlled naturalistic task could be recovered from the population activity vector of the brain. We used the video game to investigate whether representations of task variables could be recovered from brain activity during a complex and open-ended task, and also whether a data-driven method might recover these representations.

In the visual attention task (Çukur et al., 2013b), subjects viewed short natural movies while steadily fixating on a central dot. In separate runs, subjects covertly attended either to the presence of humans or the presence of vehicles in the movies. Humans and vehicles appeared in a diverse array of settings and in many different sizes, positions, and orientations. In some frames, both humans and vehicles were present. Subjects were instructed to respond with a button press when an exemplar of the attended category was present on the screen. Data for each attention condition were collected across three 10-min runs. In separate sessions, subjects passively viewed an extended set of natural movies without performing visual search. All attention conditions were mutually exclusive (i.e., there were no “attend to humans and vehicles” condition). Here we analyzed data from five subjects included in the original visual search study (Çukur et al., 2013b) as well as one additional subject whose data was acquired subsequently.

In the video game task, subjects engaged in a simulated first-person combat against computer players in *Counter Strike: Source* (Valve Co. Bellevue, WA). In this game the subject played a member of a counter-terrorism force attempting to stop a terrorist force from planting a bomb. The first subject controlled the game using two button fiber optics response pads and an MR-compatible trackwheel mouse (Current Designs, Philadelphia, PA), while the second subject used a gamepad that was modified to be MR-compatible. To alleviate the difficulty of gameplay in the scanner and to better capture game dynamics, gameplay speed was slowed down to 50% of the normal speed. Because Counter Strike: Source is an interactive open-ended game, it was not possible to obtain identical repeats of the audiovisual stimuli or player actions. Gameplay was recorded using a system built into the game engine. After the MR experiment, the audiovisual stimulus during the gameplay was reconstructed based on these recorded data. Ninety minutes of data was collected across six 15-min runs for one subject and 45 min of data was collected across three 15-min runs for the second subject. Because these data were originally collected as a pilot project, the subjects differed in the controls used and amount of time spent in the task.

#### Task Variables

The supervised state space method requires explicit definition of task variables. We therefore operationally defined task variables for each of the two tasks. Here, “task variables” can encompass both task-relevant stimulus features, and also states endogenous to the subject such as attention.

##### Visual attention task

The relevant task variables are the attentional state of the subject (attend to humans, attend to vehicles, or passive), the presence of humans in the movies, and the presence vehicles of the movies. Because the search for humans and for vehicles were performed in distinct runs, the attentional conditions were mutually exclusive. Thus, they are grouped under a single task variable. Taken together, these three variables define a three-dimensional state space that encompasses both stimulus- and task-related information.

##### Video game task

Because the video game task is complex and open-ended, ground-truth task variables are not available. To obtain reliable, human-interpretable task variables, two human observers inspected the game recordings and together they defined a set of nine behavioral states for the video game task: “dead,” “round start,” “safe explore,” “unsafe explore,” “kill,” “engage close,” “engage far,” “flash,” and “run away.” (The full definition of each state is given in Table 1). The observers then labeled each TR with these states. The states were not mutually exclusive. For this analysis, each behavioral state was treated as a task variable. Taken together, these variables define a nine-dimensional task space for the video game task that captures the behavioral state of the subject.

**TABLE 1:** Descriptions of the nine behavioral states in the video game task.

**Behavioral state**	**Definition**
Dead	The subject’s character is dead and the subject is unable to participate in the game
Round start	Prep time at the beginning of a round
Safe explore	Subject is exploring the map when it is known that the enemy is too far away to engage
Unsafe explore	Subject is exploring the map but there is a possibility of engaging enemies
Kill	Subject has killed an enemy
Engage close	Subject is engaging enemies in close quarters combat
Engage far	Subject is engaging enemies at a distance
Flash	Subject is hit with a flashbang and is unable to see
Run away	Subject is running away from enemies

*Because ground-truth task variables were not available in the open-ended video game task, two human observers inspected the game recordings and defined nine behavioral states. These states capture the range of behaviors of the subjects in the video game task and serve as human-interpretable task variables. These states were used to label each TR for the state space analyses. The states and their definitions are given here.*

#### MRI Protocols

Functional MRI data were collected using a 32-channel head coil on a 3T Siemens Tim Trio scanner at the University of California, Berkeley. Functional data were acquired using a T${}_{2}^{*}$-weighted gradient-echo EPI sequence customized with a water-excitation radiofrequency pulse to prevent contamination from fat signal. The following parameters were prescribed: repetition time = 2 s, echo time = 34 ms, flip angle = 74°, voxel size = 2.24 × 2.24 × 3.5 mm^3^, field of view = 224 × 224 mm^2^, matrix size = 100 × 100, and 32 axial slices to cover the entire cortex. Data were acquired in 325-volume runs. The first and last 10 volumes of each run were discarded. No acceleration was used. Head motion was minimized with foam padding. To enable reconstruction of cortical surfaces, anatomical data were acquired using a three-dimensional T_1_-weighted MP-RAGE sequence with the following parameters: voxel size = 1 × 1 × 1 mm^3^ and field of view = 256 × 212 × 256 mm^3^.

#### Preprocessing and Visualization

Preprocessing of functional data was performed using an in-house processing pipeline. For each subject, the Statistical Parameter Mapping toolbox (SPM8^1^) was used to align all brain volumes to the first volume in the first run. Non-brain tissues were excluded using the Brain Extraction Tool (BET)^2^. Cortical surfaces were reconstructed using Freesurfer (Dale et al., 1999). All analyses were performed on cortical voxels. All stages of preprocessing were checked carefully by hand.

Low-frequency drifts in single voxel responses were estimated using a 240-s Savistsky-Golay filter of third order and removed from the responses. The detrended voxel responses were normalized to zero mean and unit variance. No spatial smoothing or filtering was used. Functional regions of interest (ROIs) in each subject were localized using functional localizer and retinotopic mapping data collected for this purpose. Boundaries of functional ROIs were delineated on each subject’s cortex based on relative response levels to standard functional localizers (Huth et al., 2012). Mapping of functional signals to the cortical surface and visualization of results on the cortical surfaces was performed using the pyCortex toolbox (Gao et al., 2015).

It is possible that subcortical structures and the cerebellum are activated during these naturalistic tasks, particularly the video game task that requires motor commands. However, the pulse sequence used in this study was optimized for cortical signals and the slice prescription did not include the cerebellum. Therefore, the data were insufficient to permit modeling of subcortical voxels and the cerebellum, and so all analysis procedures were restricted to cortical voxels.

#### Deconvolution of the Hemodynamic Response in the Attention Task

As a further step in the preprocessing, we deconvolved the hemodynamic response function (HRF) from data in the attention task. BOLD measurements reflect delayed hemodynamic responses consequent to underlying neural activity (Ogawa et al., 1990; Kay et al., 2008a). The HRF lasts many seconds, peaking at 5–10 s from the onset (Glover, 1999). In the visual attention task, the presence of target categories can switch rapidly on the order of seconds. The HRF effectively smooths the measured brain activity and causes states between successive TRs to be less distinguishable from each other than they might be otherwise. This smoothing could hamper state space analyses. Therefore, we aimed to deconvolve the HRF from the raw brain activity by fitting a voxelwise encoding model. Briefly, voxelwise modeling (VM) (Kay et al., 2008b; Naselaris et al., 2009, 2011; Nishimoto et al., 2011) treats the activity of each voxel as a linear transformation of stimulus features. The HRF is modeled by fitting a separate finite impulse response (FIR) filter to every distinct feature separately for every voxel. The FIR filter is implemented by concatenating feature vectors that had been delayed by 1, 2, 3, and 4 TRs. In the attention task, subjects focused on the semantic contents of the stimulus. Therefore, we use the WordNet semantic category labels as used in our earlier study (Çukur et al., 2013b) as a basis for the HRF deconvolution. This model was previously shown to provide comprehensive descriptions of the responses of visual and non-visual cortical voxels to semantic information (Huth et al., 2012; Çukur et al., 2013a, 2016). To construct timecourses of the WordNet features, salient semantic categories in each 1-s movie clip were manually labeled, and then temporally downsampled to match the fMRI sampling rate. Ridge regression was used to estimate weights with 10-fold cross validation. For each voxel, the set of weights across the FIR were averaged to obtain a single weight per feature. These average weights reflect the mean selectivity of each voxel to each feature without temporal smoothing. They were then multiplied with the feature timecourses to estimate brain activity deconvolved from the HRF. This deconvolution procedure greatly improved the interpretability of state space results on the attention task.

In this study, we used existing semantic labels from previous experiments for the visual attention data. However, no such labels existed for the video game data, and only behavioral labels were available. Furthermore, it is challenging to compile an explicit feature space *a priori* that captures all aspects of the video game task. Therefore, no HRF deconvolution was applied in the video game task, and state space modeling was performed directly on measured BOLD responses. Note that, as a result, the recovered states might be less separable in the state space than if the responses had been deconvolved using an appropriate feature space.

### Voxel-Based State Space Modeling

To recover task-related state spaces from preprocessed BOLD responses, we adopt targeted dimensionality reduction, a modeling framework originally devised for primate electrophysiology experiments (Mante et al., 2013). This framework assumes that task variables are represented in the activity vector of all cortical voxels. The task-related state space is a subspace within the entire space spanned by the cortical activity vector. This subspace captures the variance in cortical activity that can be attributed to the representation of task variables. The dimensions of this task-related state space collectively reflect the representation of task variables. Each task-related brain state can then be characterized by a unique set of state variable values. The projection of the cortical activity vector into this state space characterizes the representation of state variables in the brain at each point in time. In the current paper we make no assumptions about specific brain regions that may be involved in representation of the task-related state space. Thus, all cortical voxels (voxels that fall between the pial and white matter surfaces) are analyzed in each subject (48,673 ± 4,382 voxels, mean ± std across subjects). However, this same analysis could easily be applied to specific ROIs by analyzing only the activity of voxels within those ROIs.

The voxel-based state space method consists of two steps. First, a set of task variables that are assumed to capture the underlying structure of the task are operationally defined (see section “Task Variables”). Then a low-dimensional task-related state space that is hypothesized to represent these task variables is recovered by regressing task variables on to cortical activity.

Here, cortical activity ***Y*** was modeled as a linear combination of the task variables ***X***. The timecourses were regressed against all task variable timecourses (three for the visual attention task, and nine for the video game task) using ordinary least squares (OLS) regression. We use OLS instead of ridge regression here because the number of state variables is far fewer than the number of samples, and thus overfitting is not likely to be a concern. This step is identical to traditional univariate analyses of voxelwise regression with binary categorical features. This procedure yielded a set of weights ***B*** that map task variables to cortical activity [size (variables × voxels)] ***(Y* = *XB)***.

The set of weights obtained for each task variable forms a vector of length (voxels) in the cortical activity space. Each weight vector represents the characteristic pattern of cortical activity associated with the representation of that task variable. However, these weights are inherently noisy since they can implicitly capture other non-task-related activity patterns irrelevant to this experiment and which account for little variance. To focus on the task-driven activity, a denoiser was created using principal component analysis (PCA) as was performed in the original electrophysiology analysis method (Mante et al., 2013). PCA was performed on the timecourse ***Y*** of cortical activity [size (TRs × voxels)] in individual subjects. The first 24 eigenvectors of ***Y***, ***U*_L_**, were retained [size (voxels × 24)], accounting for ∼70% of the total variance in BOLD responses, and a denoiser ***D*** was calculated as ***D* = *U_L_ U_L_^*T*^***.

Next, ***B*** was denoised using ***D*** to create ***B_L_* = *DB*** and to form the state space. Note that the weight vectors of the task variables are not guaranteed to be orthogonal. To form an orthonormal basis for the task-related state space, QR decomposition was performed on the transpose of ***B*_L_** [size (variables × voxels)], factorizing ***B_L_^*T*^* = *QR***. The first (variables) columns of the resulting ***Q*** [size (voxels × voxels)] matrix, ***Q*_S_**, then represent the dimensions of the state space. Each dimension in this state space reflects the brain’s representation of one task variable. Finally, the cortical activity vector at each TR was projected to a point in this state space ***(p_i_* = *y_i_Q_S_)***. This point is the estimate of the state of the brain at that TR.

### Quantification of State Space Results

We developed two convergent metrics to validate the performance of the state space model. The first metric is based on the assumption that a good state space should allow clear separation of different states. To quantify this we defined a cluster separation index (CSI) as follows. First, a multivariate normal distribution was fitted to each cluster. Next, all pairwise Jensen-Shannon divergences (JSD) between the clusters were calculated (Lin, 1991). JSD provides a measure of the difference between two distributions by averaging the Kullback-Leibler divergence (KLD) (Kullback and Leibler, 1951) of individual distributions from their average distribution. We chose the JSD because it is symmetric and has a finite upper bound. Finally, the CSI was taken as the average of all inter-cluster JSDs. CSI ranges from 0 to 1, where 0 indicates no separation and 1 indicates complete separation. While visualization occurs in 2D space, CSI values were calculated in the original state space (3 dimensions for the visual attention task and 9 dimensions for the video game task).

The second metric is based on the assumption that any valid state space model should accurately predict state given new cortical activity. To quantify this we used a 10-fold cross-validation procedure to evaluate state-space prediction performance in each individual subject. In each fold, 10% of the data were held out as the validation set, and the proposed method was applied on the remaining portion to learn the state space. Each TR in the training set was labeled using the same labels used for the supervised method. The visual attention task used 12 labels (the four labels for human and vehicle presence × three attention conditions), while the video game task used nine labels (the set of behavioral states). Afterward, each TR in the validation set was projected into the learned space, and assigned to the nearest cluster to classify its state. This label was then compared against the ground truth label. Classification accuracy was averaged across cross-validation folds. For both metrics, significance was evaluated using permutation tests. Null models were created by permuting the state labels, and performing the full analysis using the permuted labels.

## Results

To understand how task information may be represented in the population activity space of the cortex, we developed a voxel-based state space framework for fMRI. The task-related state space is recovered by regressing task variables directly on to cortical activity, and activity at each TR can then be projected to a point in this task-related state space. We use this framework to recover low-dimensional task-related state spaces from cortical activity recorded during a visual attention task and a video game task.

### The Voxel-Based State Space Method Recovers a State Space for Variables in the Attention Task

**FIGURE 1:**
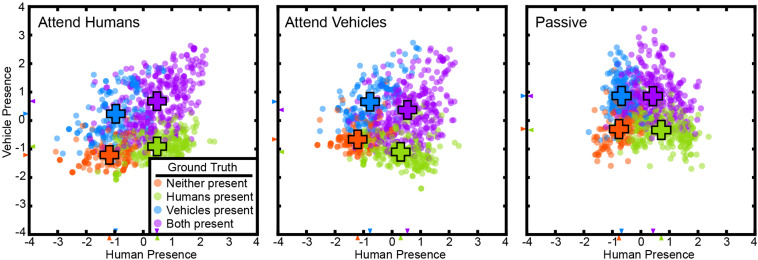
The state space method recovered a task-related state space in the visual attention task. Cortical activity at each TR for subject S2 is projected to a point in this space. The state space is projected on to the 2D plane spanned by the “human presence” (horizontal) and “vehicle presence” (vertical) axes. Projections are shown for the attend-human, attend-vehicles, and passive viewing conditions. Positive values indicate presence, and negative values indicate absence. TRs are color-coded by their ground truth states. Orange: neither present; blue: only humans present; green: only vehicles present; purple: both humans and vehicles present. Crosses indicate the mean positions of each group. The cluster separation index (CSI) is 0.75 for attend-humans, 0.69 for attend-vehicles, and 0.67 for passive (*p* < 1e-5, permutation test), all indicating significant separation of the clusters. Clusters are more distinct in the attentive conditions than in the passive condition (*p* < 1e-5, permutation test). These results are consistent with the hypothesis that task variables are represented in a low-dimensional task-related subspace of the cortical activity space, and that attention increases the separation of the states in this task-related state space.

In the attention task, subjects watched short, naturalistic movies while attending to the presence of humans or vehicles in different runs, or they watched the movies passively. The visual attention task had three main variables: the presence of humans, the presence of vehicles, and the attention target of the subject. We applied the voxel-based state space method to data from this task to examine whether task variables are represented in the cortical activity vector. The voxel-based state space method was used to learn a state space on data from all attentional conditions for each subject separately. Figure 1 shows the recovered state space for one subject, projected onto the 2D plane defined by human- and vehicle-presence axes (for all subjects see Supplementary Figures 1, 2). On this plane, TRs were labeled with the states of “only humans present,” “only vehicles present,” “neither present,” and “both present.”

Animal studies show neural activities corresponding to different choices projected to distinct regions in their state space (Harvey et al., 2012; Mante et al., 2013; Vedder et al., 2017). Based on this result we hypothesize that a good state space should allow clear separation of different states. Therefore, the state space was evaluated by measuring how well these states were separated. A CSI was defined as the average pairwise JSD of the state clusters. The states are distinctly separated with a CSI of 0.66 ± 0.09 (mean ± std across subjects) for attend-humans, 0.74 ± 0.03 for attend-vehicles, and 0.53 ± 0.24 for passive viewing (*p* < 1e-5; permutation test). These results show that task variables are represented in a low-dimensional task-related state space that can be recovered successfully by the supervised state space method. Furthermore, we found that the CSI in the passive condition was significantly lower than the CSI in the attentive conditions for five out of the six subjects (*p* < 1e-5; permutation test).

Any valid state space model should accurately predict brain states in a new data set that was not used to fit the model. To test this, cross-validated (10-fold) classification performance in each subject was evaluated using 12 distinct labels (the four labels for human and vehicle presence × three attention conditions). Average performance across subjects is 48.4 ± 7.6% (*p* < 1e-5, permutation test, chance = 8.3%). This result indicates that the recovered state space can be used to predict cortical state in a separate test data set.

**FIGURE 2:**
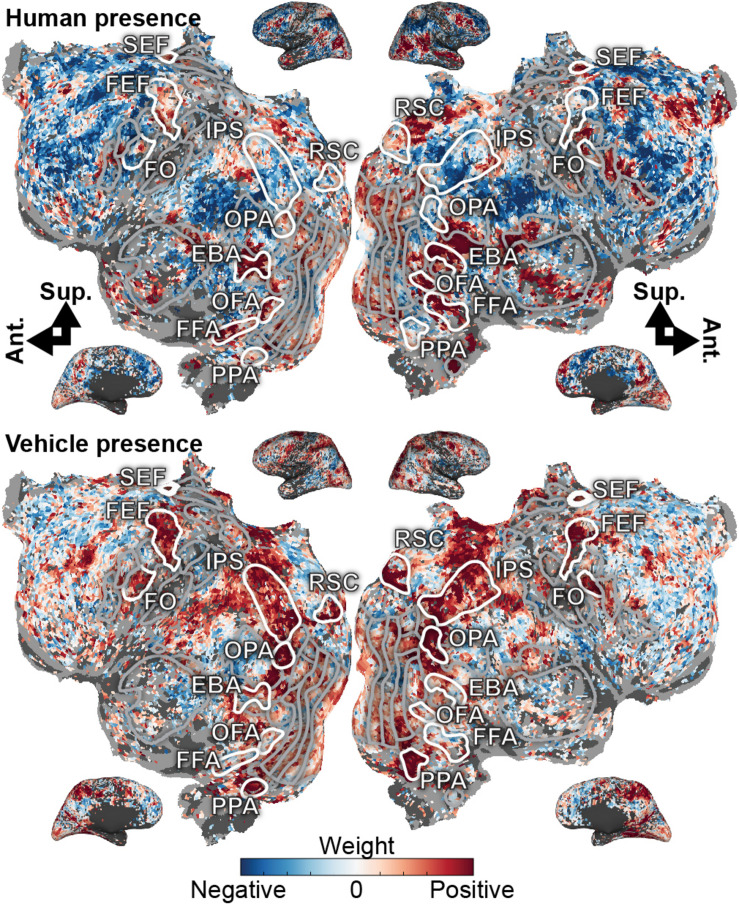
Task variables in the visual attention task are represented in broad functionally specialized networks distributed across the cerebral cortex. Model weights for human- and vehicle-presence are shown on the flattened cortical surface for subject S1. Blue correspond to negative weights, white to no weights, and red to positive weights. Voxels are thresholded by the prediction performance of a semantic encoding model at a non-FDR-corrected *p* < 0.05 significance level. Selected ROIs are highlighted and labeled. Inflated cortices are shown for reference. The human presence axis is represented in FFA, OFA, and EBA, which are known to be activated by faces or body parts. Additionally, it is represented in MT, LO, SEF, and around the inferior frontal sulcus. The vehicle presence axis is represented in RSC, OPA, and PPA, all activated during spatial perception or navigation; in the IPS and FEF, both involved in spatial attention; and also in FO and inferior frontal sulcus. These model weights agree well with the known functions of the cortex and further suggest that task variables are meaningfully represented in a low-dimensional subspace of cortical activity.

To better understand the cortical representations of the recovered state space, we projected the weights for each dimension of the task-related subspace onto the cortical surface (Figure 2 and Supplementary Figure 3). The “human presence” dimension projects heavily onto FFA (Kanwisher et al., 1997), OFA (Gauthier et al., 2000), and EBA (Downing et al., 2001), functional areas that represent faces and/or body parts. This dimension also projects onto MT+ (Zeki et al., 1991; Tootell et al., 1995), which represents motion and human movement, LO (Grill-Spector et al., 1999), which is activated during object perception, and the frontal operculum (FO) (Corbetta et al., 1998), activated in spatial attention tasks. The “human presence” dimension also projects to the inferior frontal sulcus, which is known to contain a face-selective region (Avidan et al., 2005; Tsao and Livingstone, 2008). In contrast, the “vehicle presence” dimension projects strongly onto RSC (Maguire, 2001; Epstein, 2008), PPA (Epstein et al., 1999), and OPA (Dilks et al., 2013), regions known to be active during spatial perception or navigation. The “vehicle presence” dimension also projects onto the IPS (Kastner et al., 1999; Silver et al., 2005) and frontal eye field (FEF) (Paus, 1996; Corbetta et al., 1998; Kastner et al., 1999; Moore and Fallah, 2001), both of which are activated in tasks requiring spatial attention. Finally, the “vehicle presence” dimension projects around the inferior frontal sulcus. These results show that the dimensions of the task-related state space recovered by the voxel-based state space method are related systematically to the functional selectivity of the brain regions that are activated under each state.

### The Voxel-Based State Space Method Recovers a Behavioral State Space for the Open-Ended Video Game Task

Because the visual attention task involves explicit task states, it is relatively straightforward to perform a state space analysis of those data. It is more difficult to perform a state space analysis of complex, open-ended tasks. To explore this issue, we applied the supervised method to a video game task. In this task, subjects engaged in simulated combat in *Counter Strike: Source*. Each TR was labeled using nine experimenter-defined behavioral states (Table 1). These were used as task variables for the supervised method. Because it is impractical to fully decorrelate the multitude of states that arise during complex naturalistic tasks, these states are partially correlated. Thus, this analysis also serves to test whether the state space method can recover a task-related state space even when states are correlated.

**FIGURE 3:**
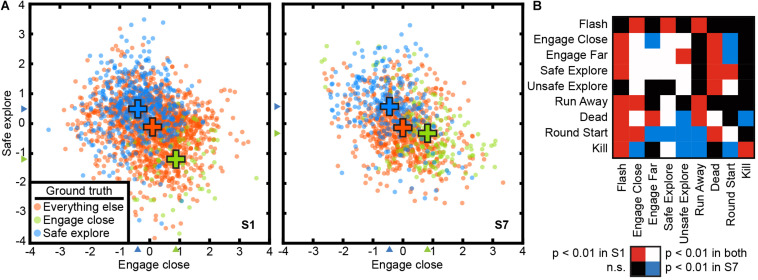
The state space method recovered a task-related state space in the open-ended video game task. Cortical activity at each TR is projected to a point in a 9-dimensional state space. The state space method revealed significant relationships between the nine behavioral states in the video game task. The nine states are not orthogonal, and some states are likely to lead to others. **(A)** The state space projected to two dimensions for “safe explore” and “engage close” for both subjects. TRs are color-coded by their ground truth state. Blue: safe explore; Green: engage close; Orange: all other states. Crosses indicate the mean positions of each group. The CSI was 0.72 for subject C1 and 0.62 for subject C2, indicating significant separation of the clusters (*p* < 1e-5, permutation test). These two states are mutually exclusive and score negatively on each other’s axis. **(B)** Relationships between states are reflected in non-zero projections of states on to other state dimensions. Rows and columns are behavioral states as described in Table 1. The table is asymmetric because relationships between states are not necessarily reciprocal. Colors indicate Bonferroni-corrected significance (*p* < 0.01, permutation test). Red: significant for subject C1; Blue: significant for subject C2; White: significant in both subjects; Black: not significant in either subject. Because the task is open-ended, subjects may adopt different strategies and thus the exact pattern of correlation differs between subjects. Task variables in a complex, open-ended task are represented in a low-dimensional space in cortical activity that also captures relationships between states.

A 9-dimensional task-related state space was learned for the video game task for each subject using the voxel-based state space method. Figure 3 shows two dimensions of the recovered state space for both subjects, projected onto two planes each defined by “engage close” and “safe explore.” These two dimensions were chosen as the corresponding brain states project not only on to their own dimension, but also on to the other. States are labeled using the nine behavioral labels shown in Table 1. The states are distinctly separated with a CSI of 0.72 in subject S1 and 0.62 in subject S7 (*p* < 1e-5, permutation test). Thus, task variables appear to be represented in a low-dimensional space even in this complex, open-ended task.

To gain a better understanding of how state correlations affect these low-dimensional task representations in brain activity, we examined the projection of states on to other dimensions in the task-related state space. We find that when states are correlated, TRs associated with one state project to a non-zero position in the dimension associated with the other state. For example, “engaging close” and “safe explore” each project negatively to the dimension associated with the other state (Figure 3A) (*p* < 1e-5, permutation test). The full list of significant correlations between states for the two subjects is given in Figure 3B. The exact patterns of correlations differ between subjects; because the video game task is open-ended, the subjects used different strategies and these resulted in different game dynamics and different correlations. Nonetheless, the correlations between states are captured by the low-dimensional representations in the brain activity of both subjects.

As noted earlier, any valid state space model should accurately predict brain states in a new data set that was not used to fit the model. We therefore performed a separate test to determine whether the state space can be used to predict cortical state in the video game task. Because the video game task is open-ended, the behavioral states are not all equally like to occur. Thus, a simple accuracy score may misrepresent prediction accuracy by, for example, simply always guessing the most common class. We therefore used the balanced prediction accuracy metric (Brodersen et al., 2010), which accounts for the unequal frequency of classes by averaging the recall score on each class. Average balanced accuracy across 10 cross-validation folds in the subject is 32.8 ± 3.8% in subject S1 and 30.4 ± 4.2% in subject S7 (*p* < 1e-5, permutation test, chance = 11%). This result suggests that the learned state space can be used to predict cortical state even in the presence of correlations between states.

**FIGURE 4:**
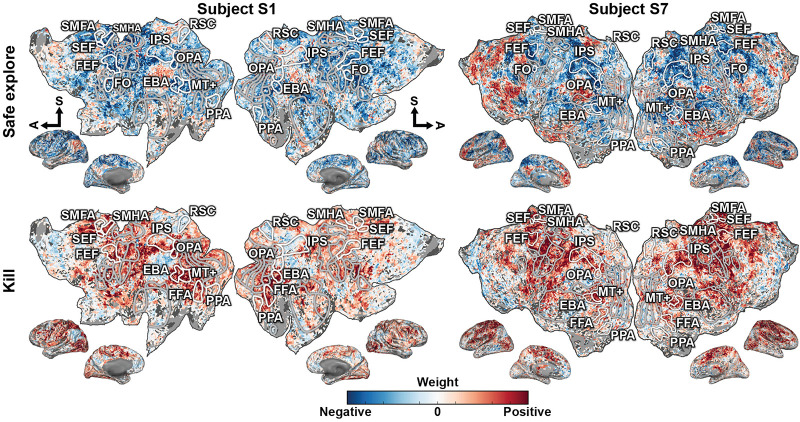
Behavioral states in the video game task are represented in broad functionally specialized networks distributed across the cerebral cortex. The representations fall into two general groups that share overlapping regions. Weights for one state dimension from each group, “safe explore” and “kill,” are shown on the flattened cortical surfaces for both subjects. Blue correspond to negative weights, white to no weights, and red to positive weights. Voxels are thresholded by the prediction performance of an encoding model at a Bonferroni-corrected *p* < 0.05 significance level. Selected ROIs are highlighted and labeled. Inflated cortices are shown for reference. The axes for “dead,” “safe explore,” “round start,” “flash,” and “unsafe explore” share representation in the TPJ, precuneus, and prefrontal cortex. The axes for “kill,” “engage close,” “run away,” and “engage far” share representation in the motor, pre-motor, and supplementary motors areas, and also in IPS, FEF, and SEF. These model weights further suggest that task variables in complex and open-ended tasks are also represented in a low-dimensional subspace of cortical activity.

To understand how the recovered states are represented in the brain, the model weights for each dimension were projected onto the cortical surface for both subjects (Figure 4 shows two dimensions for both subjects, for all dimensions see Supplementary Figures 4, 5). They appear to fall into two general categories. The dimensions for “safe explore,” “round start,” “flash,” “dead,” and “unsafe explore” project on to the TPJ, precuneus, and prefrontal cortex. The dimensions for “run away,” “engage close,” “kill,” and “engage far” project on to the motor, pre-motor, and supplementary motors areas, and also in IPS, FEF, and SEF. Because the specific task states examined here have not been investigated in previous studies, it is difficult to relate these functional assignments to neuroimaging literature. However, it appears that the first category predominantly engages areas within the default-mode network, while the second category predominantly engages areas that represent selective attention and motor movement. The weights for each dimension are largely consistent across the two subjects. These results suggest that there is a low-dimension representation of task variables in cortical activity even in more complex and open-ended tasks.

## Discussion

Complex naturalistic behaviors evoke complex, high-dimensional activity across the cortex that are challenging to analyze and interpret (Hasson et al., 2004; Mathiak and Weber, 2006; Spiers and Maguire, 2007; Mathiak et al., 2011; Nishimoto et al., 2011; Huth et al., 2012, 2016; Çukur et al., 2013b). To understand this complex activity, we developed a voxel-base state space modeling framework to find interpretable, low-dimensional representations of task variables. In both a visual attention task and a video game task, we find a low-dimensional representation of task variables in cortical activity. Furthermore, the cortical areas associated with representations during the attention task agree well with previous studies.

These low-dimensional representations also capture both attentional effects on representation in the attention task and correlations between representations in the video game task. In previous studies (David et al., 2008; Çukur et al., 2013b) we found that attention warps semantic representations by increasing the distance between categories in semantic space. Here, the CSI in the passive condition was significantly lower than the CSI in the attentive conditions in all but one subject. Thus, attention to semantic categories significantly increases the separation of states in the task-related subspace relative to what is found under passive viewing. The increased separation in state space directly reflects attentional warping: both serve to increase the cortical representations of task-relevant variables to facilitate the task. This finding agrees with other studies that also found attention improved representation of features, such as color (Brouwer and Heeger, 2013), physical and conceptual properties (Harel et al., 2014), or animal taxonomy and behavior (Nastase et al., 2017). Unlike these prior studies, here we were able to demonstrate the increased separation directly in the activity space of the brain and using much more naturalistic stimuli. Thus, these data provide further evidence that attention warps functional representations to improve task performance (David et al., 2008; Çukur et al., 2013b). In the video game task, the behavioral states could not be counterbalanced and therefore were not independent of each other. In the task-related state space, we find that representations of correlated behavioral states are not orthogonal. Correlated states project to non-zero points on each other’s dimension in state space, showing correlated task variables have similar cortical representations.

There are several limitations concerning the task-related state space we recovered from the rich video game data. First, we used nine behavioral labels for the state space data that were hand-picked by observers. While the labels were agreed upon between two observers familiar with the video game, different observers might pick different labels and thus would produce a different state space. Second, behavioral labels can only serve as a proxy to true task variables. An objective parameterization of the video game task would produce a better set of variables from which to build the task-related state space. Using machine learning to parametrize the game state may be one such avenue for future research (Jaderberg et al., 2019). Alternatively, a completely unsupervised method may be able to recover a task-related state space in a completely data-driven manner. However, using an unsupervised method would raise the issue of interpreting the recovered state spaces in the context of the tasks. Last, we collected only a very limited data set using the video game task. Therefore, the data may have simply been too noisy to allow us to recover the optimal task-related state space from cortical data. Nevertheless, the current results support the existence of low-dimensional representations even in complex and open-ended tasks, and suggest that future work focusing on the video game task is promising.

Many other methods have been proposed for modeling high-dimensional cortical activity. Multivoxel pattern analysis (MVPA) (Haxby et al., 2001; Cox and Savoy, 2003; Kamitani and Tong, 2005; Norman et al., 2006) decodes the stimulus or task from the activity of a set of voxels to provide a categorical description of brain activity. The classifier used to evaluate the quality of the state space model is mathematically similar to MVPA in that some stimulus or task variable is decoded from the activity of a collection of voxels. However, the state space method provides a model of how task variables are continuously encoded in the cortical activity vector of the brain. The classifier is distinct from this model and does not form the core of the state space method. The classifier is not used to interpret the recovered state space, and is used only to evaluate the quality and stability of the recovered state space. A few studies (Billings et al., 2017; Shine et al., 2019a, b) have also attempted to recover low-dimensional cortical activity in data from the Human Connectome Project (HCP) (Van Essen et al., 2013). However, because the HCP used multiple tasks and collected extremely limited amount of data per task, these studies can only examine the general activity differences between tasks. They cannot describe how variables within each task are represented in cortical activity.

In sum, our results suggest that while overall cortical activity is high-dimensional, the representation of particular task variables is reflected in a low-dimensional subspace of brain activity. Analyzing the cortical activity vector of the brain complements analyses on single voxel activities by revealing the complex interactions between brain systems. These interactions can be useful for discovering functionally linked networks of brain regions. We expect the voxel-based state space method will be useful in understanding high-dimensional brain activity elicited by complex, open-ended naturalistic tasks.

## Data Availability Statement

The data is publicly available at CRCNS at https://dx.doi.org/10.6080/K0668BDF and the code is available on Github at https://github.com/gallantlab/state-space.

## Ethics Statement

The studies involving human participants were reviewed and approved by the Committee for Protection of Human Subjects, University of California, Berkeley. The patients/participants provided their written informed consent to participate in this study.

## Author Contributions

TZ, JSG, TÇ, and JLG designed the research. JSG and TÇ performed the research. TZ analyzed the data, wrote the first draft of the manuscript, and wrote the manuscript. TZ, TÇ, and JLG edited the manuscript. All authors contributed to the article and approved the submitted version.

## Conflict of Interest

The authors declare that the research was conducted in the absence of any commercial or financial relationships that could be construed as a potential conflict of interest.

## Footnotes

1 http://www.fil.ion.ucl.ac.uk/spm/software/spm8/

2 http://fsl.fmrib.ox.ac.uk/fsl/fslwiki/BET
