# Supplementary material for: Voxel-Based State Space Modeling Recovers Task-Related Cognitive States in Naturalistic fMRI Experiments
Source: Front Neurosci. 2021 May 6;14:565976. doi: 10.3389/fnins.2020.565976 (PMC8145286; doi:10.3389/fnins.2020.565976)
Supplement: Supplementary file 1 [file Data_Sheet_1.DOCX]

Supplementary Material

# Supplementary Figures

**
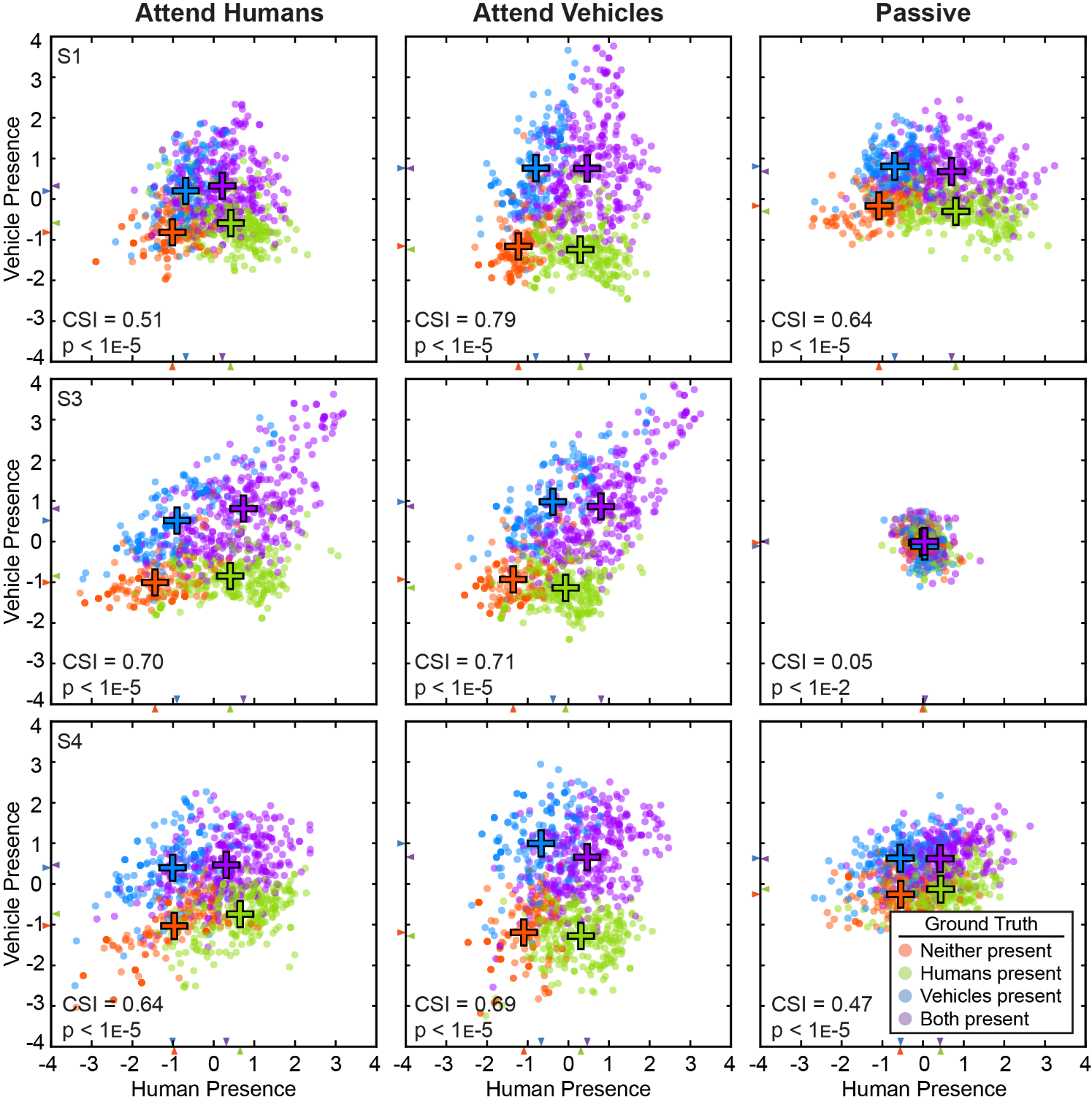
Supplementary Figure 1.** A task-related state space for subjects S1, S3, and S4 in the visual attention task. A task-related state space is recovered separately for each subject. Cortical activity at each TR for subject is projected to a point in that subject’s space. The state space is projected on to the 2D plane spanned by the “human presence” (horizontal) and “vehicle presence” (vertical) axes. Each row corresponds to the state space for one subject. Projections are shown for the attend-human (left column), attend-vehicles (center column), and passive (right column**)** viewing conditions. Positive values indicate presence, and negative values indicate absence. TRs are color-coded by their ground truth states. Orange: neither present; blue: only humans present; green: only vehicles present; purple: both humans and vehicles present. Crosses indicate the mean positions of each group. CSIs are shows for each condition in each subject and are significant (p < 1e-2 or less, permutation test). Clusters are more distinct in the attentive conditions than in the passive condition (p < 1e-5, permutation test). These results are consistent with the hypothesis that task variables are represented in a low-dimensional task-related subspace of the cortical activity space, and that attention increases the separation of the states in this task-related state space.


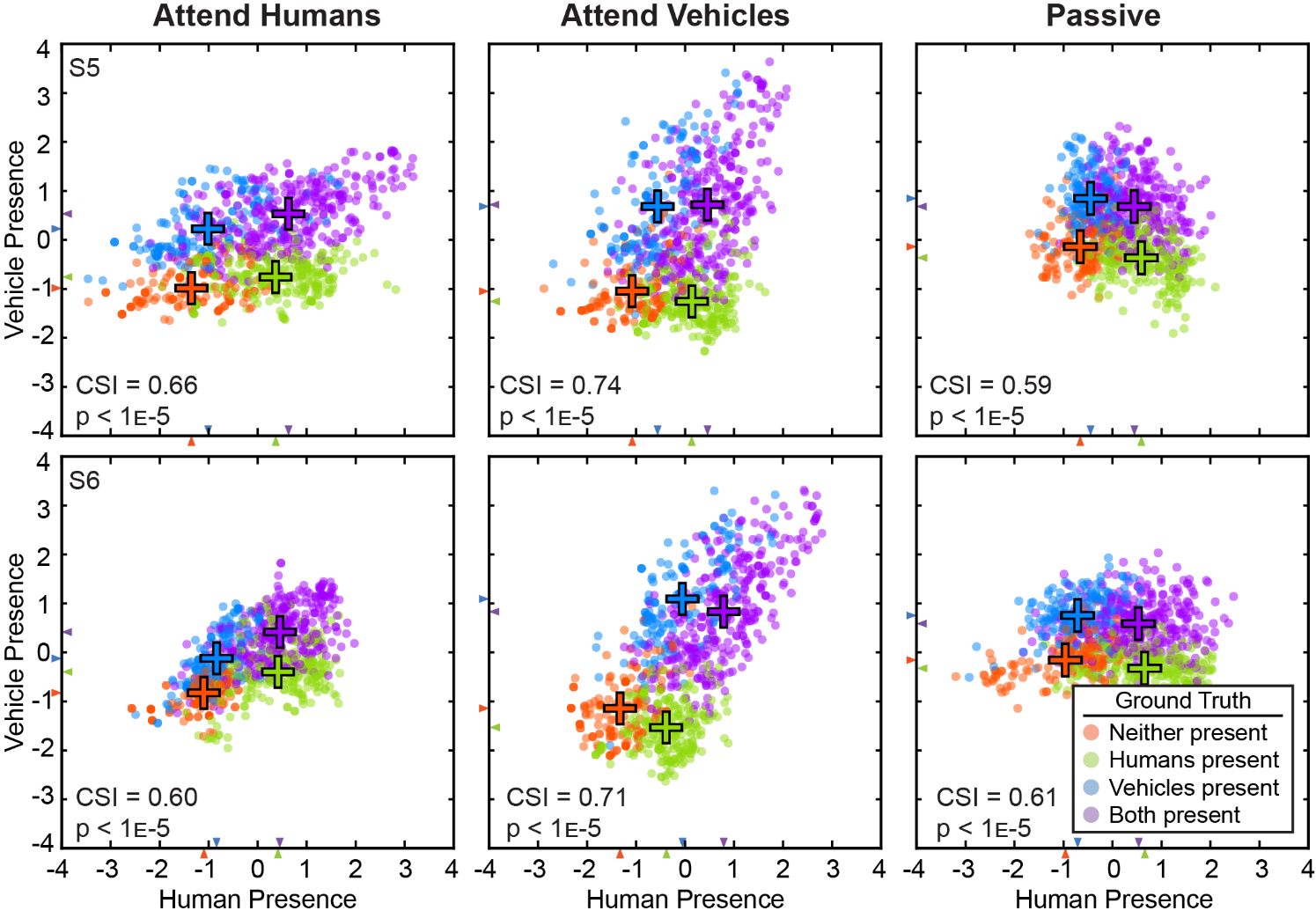


**Supplementary Figure 2.** A task-related state space for subjects S5 and S6 in the visual attention task. A task-related state space is recovered separately for each subject. Conventions are the same as in supplementary figure 1.


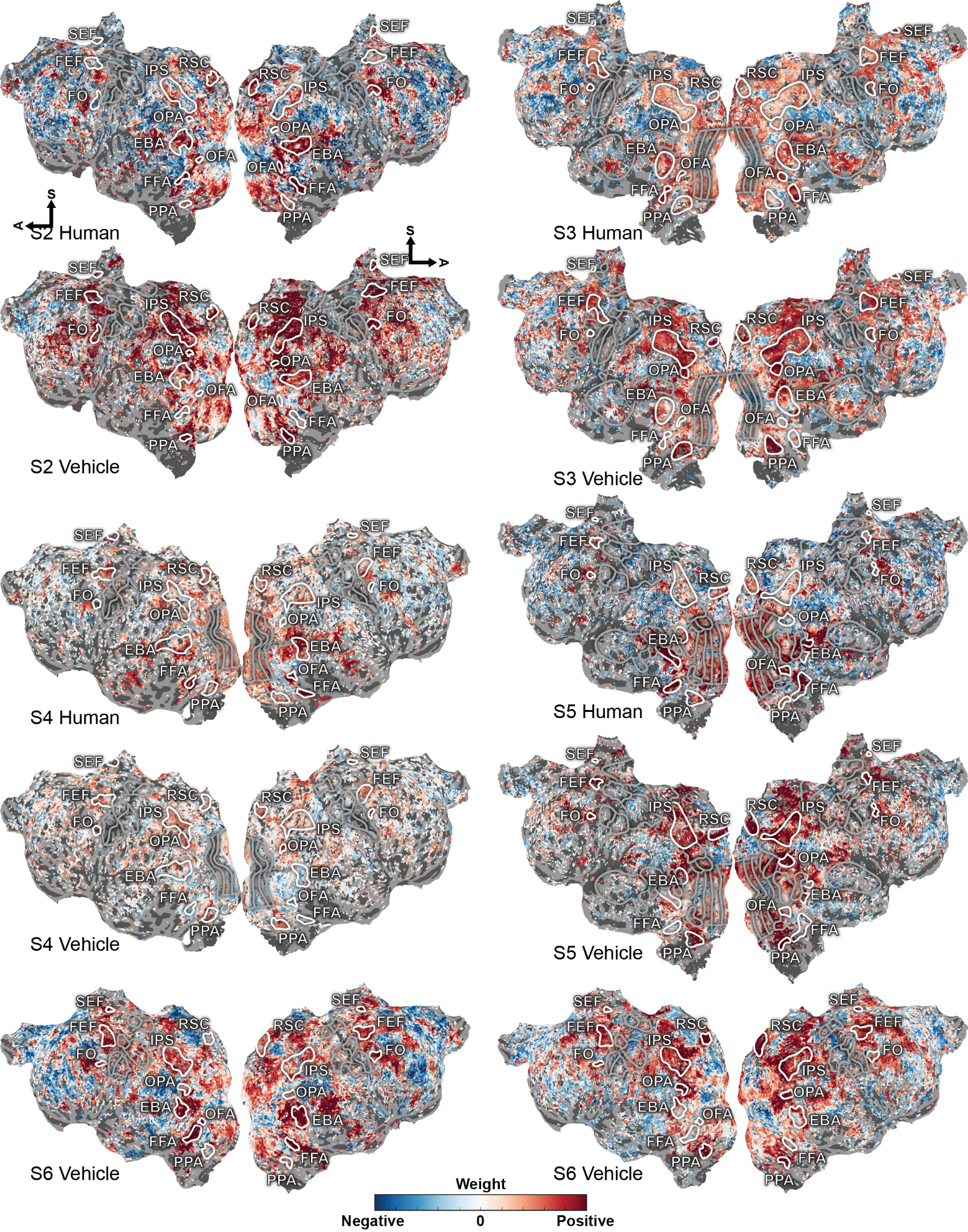


**Supplementary Figure 3.** Task variables in the visual attention task are represented in broad functionally specialized networks distributed across the cerebral cortex. Model weights for human- and vehicle-presence are shown on the flattened cortical surface for subjects S2-S6. Blue correspond to negative weights, white to no weights, and red to positive weights. Voxels are thresholded by the prediction performance of a semantic encoding model at a non-FDR-corrected p < 0.05 significance level. Selected ROIs are highlighted and labelled. These model weights agree well with the known functions of the cortex and further suggest that task variables are meaningfully represented in a low-dimensional subspace of cortical activity.


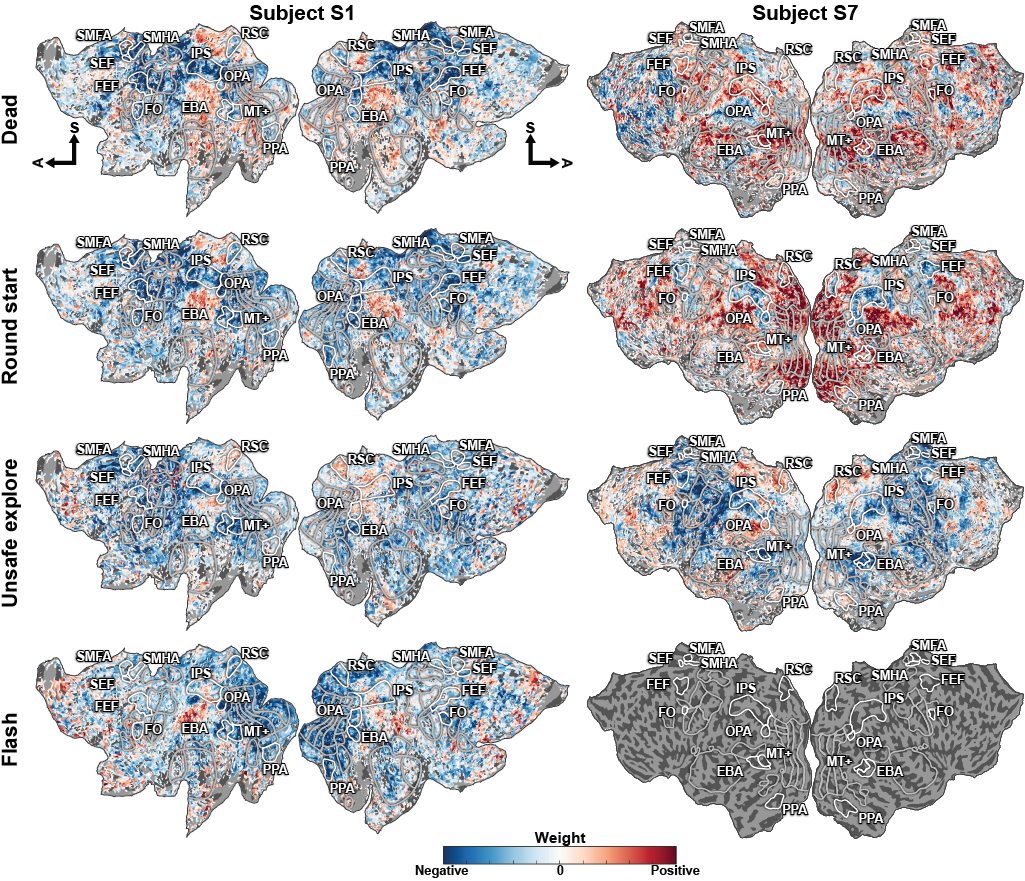


**Supplementary Figure 4.** Behavioral states in the video game task are represented in broad functionally specialized networks distributed across the cerebral cortex. Weights for “dead”, “round start”, “unsafe explore”, and “flash” are shown on the flattened cortical surfaces for both subjects. Blue correspond to negative weights, white to no weights, and red to positive weights. Voxels are thresholded by the prediction performance of an encoding model at a Bonferroni-corrected p < 0.05 significance level. Selected ROIs are highlighted and labelled. These dimensions share representation in the TPJ, precuneus, and prefrontal cortex. Note that there are no weights for “flash” for subject C2, because C2 did not experience any “flash” events during the video game task.


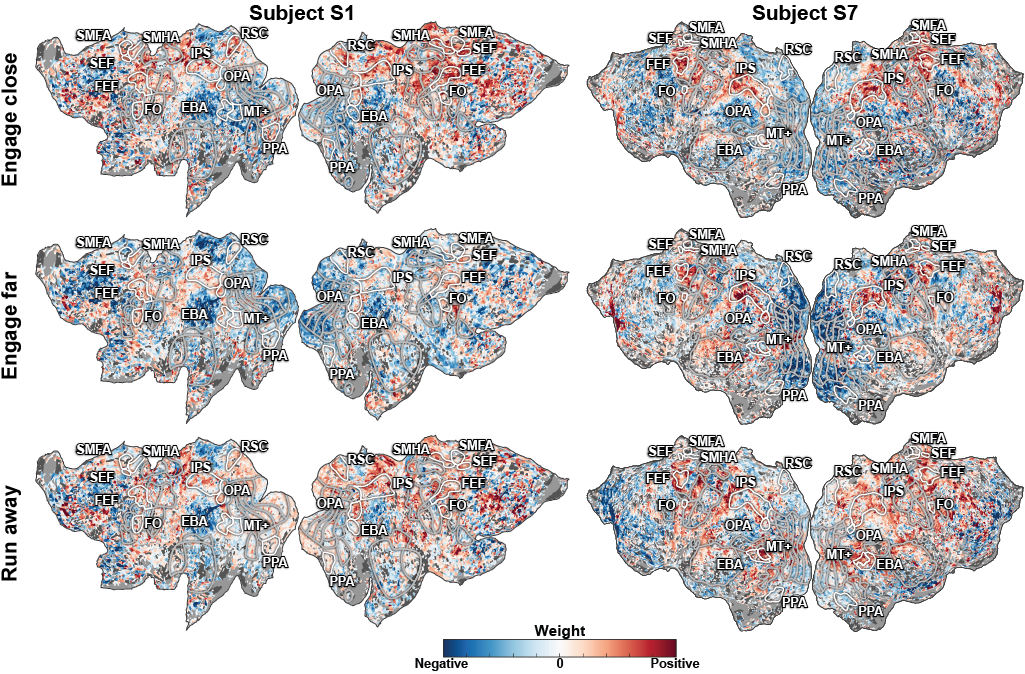


**Supplemental Figure 5**. Behavioral states in the video game task are represented in broad functionally specialized networks distributed across the cerebral cortex. Weights for “engage close”, “engage far”, and “run away” are shown on the flattened cortical surfaces for both subjects. Blue correspond to negative weights, white to no weights, and red to positive weights. Voxels are thresholded by the prediction performance of an encoding model at a Bonferroni-corrected p < 0.05 significance level. Selected ROIs are highlighted and labelled. These dimensions share representation in the in the motor, pre-motor, and supplementary motors areas, and also in IPS, FEF, and SEF.
